# The effect of anti-dementia drugs on Alzheimer disease-induced cognitive impairment: A network meta-analysis

**DOI:** 10.1097/MD.0000000000016091

**Published:** 2019-07-05

**Authors:** Cui-Cui Cui, Yong Sun, Xin-Yi Wang, Yuan Zhang, Ying Xing

**Affiliations:** China-Japan Union Hospital of Jilin University, Changchun, China.

**Keywords:** Alzheimer disease, cognitive disorders, dementia, MMSE, network meta-analysis

## Abstract

**Background:**

Cognitive impairment is a principal manifestation of Alzheimer disease (AD). To provide a clinical reference for the treatment of AD, a network meta-analysis (NMA) was performed to evaluate the effects of different anti-dementia drugs on the cognitive impairment exhibited by patients with AD.

**Methods:**

Relevant randomized controlled trials are found through the Pubmed database, Web of Science, Clinical Trials, Embase, Cohranne library, Chinese National Knowledge Infrastructure database, CBM databases, and Wanfang among others. A total of 33 articles were collected, with the earliest document collected having been published in February 2017. The included reports were screened for quality of papers by using strict inclusion and exclusion criteria. All analyses were based on previously published studies reporting de-identified data; thus, no ethical approval or patient consent were required. The Mini-Mental State Examination scores informed the classification of the 33 articles into a mild subgroup, which featured 11 articles, and 12 drugs (besides a placebo); a moderate subgroup, which featured 17 articles and 15 drugs (besides a placebo); and a severe subgroup, which featured 5 articles and 3 drugs (besides a placebo).

**Results:**

While donepezil, galanthamine, and huperzine demonstrated the highest efficacy in the mild cognitive dysfunction subgroup (mean difference = 5.2, 2.5, and 2.4, respectively). Donepezil, huperzine A, and rivastigmine achieved the most significant effects in the moderate cognitive dysfunction subgroup (MD = 3.8, 2.9, and 3.0 respectively). In the severe subgroup, donepezil was demonstrably superior to memantine. Donepezil was thus found to effectively address cognitive impairment in patients with AD regardless of the degrees of cognitive decline.

**Conclusions:**

Evaluation of the clinically common anti-dementia drugs using NMA affirmed the utility of cholinesterase inhibitors, especially donepezil, in alleviating cognitive dysfunction of patients with AD. This study may therefore help to inform the clinical selection of pharmacotherapeutic interventions addressing cognitive dysfunction in patients with AD.

## 1 Introduction

Cognitive impairment is one of the main manifestations of Alzheimer disease (AD), and the efficacy of anti-dementia drugs is directly related to its therapeutic attenuation of the cognitive impairments and prognosis of patients with AD. The present study evaluated the outcomes of different anti-dementia drugs on the cognitive function of patients with AD through network meta-analysis (NMA). Pubmed databases, Web of Science, Clinicaltrials, Embase, the Cohranne library, the Chinese National Knowledge Infrastructure database (CNKI), CBM databases, and Wanfang databases among others were systematically searched for eligible randomized controlled trials (RCTs). The retrieved reports were screened for quality according to our strict inclusion and exclusion criteria, yielding a total of 33 articles. The MMSE scale informed the classification of the 33 articles into a mild subgroup, which featured 11 articles and 12 drugs (besides a placebo); a moderate subgroup, which featured 17 articles and 15 drugs (besides a placebo); and a severe subgroup, which featured 5 articles and 3 drugs (besides a placebo). We found that donepezil, galanthamine, and huperzine achieved the most significant effects in the mild cognitive dysfunction subgroup (mean difference (MD) = 5.2, 2.5, 2.4, respectively). In the moderate cognitive dysfunction subgroup, donepezil, huperzine A, and rivastigmine achieved the most significant effects (mean difference = 3.8, 2.9, 3.0, respectively). In the severe subgroup, donepezil was demonstrably superior to memantine. This analysis therefore demonstrated the cognitive impairment-independent efficacy of donepezil in treating patients with AD.

AD is a degenerative disease of the central nervous system that occurs in presenium. The clinical manifestations of AD include memory disorders, aphasia, apraxia, agnosia, visuospatial disability, compromised abstract thinking, and computational power, as well as personality and behavioral disorders. These symptoms deteriorate progressively, eventually causing the complete loss of motor function.^[1]^ Although there is no specific treatment to reverse or prevent the progression of AD, treatment strategies that provide early support and symptomatic relief can mitigate the rapid decline in the patients’ quality of daily life. In order to provide a clinical reference for the clinical treatment of AD, the present study performed a network meta-analysis (NMA) to evaluate the effect of different anti-dementia drugs on the attenuation of cognitive function impairment of patients with AD.

## 2 Data and methods

### 2.1 Inclusion and exclusion criteria

#### 2.1.1 Inclusion criteria

1. Randomized controlled trials (RCTs) of AD.
2. Inclusion of patients with different degrees of AD.
3. Experimental interventions included different anti-dementia drugs, and a placebo was administered to the control group.
4. Inclusion of a merge-control group: experimental intervention administered at different doses.
5. Average Mini-Mental State Examination (MMSE) scores were used to evaluate study outcomes.

#### 2.1.2 Exclusion criteria

1. The reports were either a non-RCT, letter, editorial, commentary, or case report.
2. No definite curative effect was assessed, or curative effects were assessed for less than 3 months.
3. Inclusion of subjects with vascular dementia; Parkinson disease-induced dementia; delirium; depression and other mental disorders; congenital brain function hypoplasia, such as Down syndrome; or subarachnoid hemorrhage.

### 2.2 Data sources and retrieval method

All relevant RCTs were selected through the Pubmed database, Web of Science, Clinical Trials, Embase, Cohranne library, Chinese National Knowledge Infrastructure database, CBM databases, and Wanfang databases among others.AD, cognitive impairment, or dementia were used in all databases – except Embase – as keywords for retrieval. Terms from the MESH subject word list provided by Medline were used to retrieve studies from foreign databases. In addition to using Disorder, Cognition, Disorders, Dysfunction, and Huperzine A, Donepezil, Rivastigmine, Galantamine, Idebenone, Vitamin E, N-Acetylcysteine, Estrogen, Melatonin, Folic Acid, and Statins were used as keywords or uncontrolled terms in both Chinese and English to retrieve suitable studies. In order to comprehensively collect information relevant to our study objective, we did not exclude studies based on their language or their date of publication so long as they were randomized controlled trials. The earliest retrieved study was performed in February 2017.

### 2.3 Quality evaluation

All following data was extracted from the eligible studies by 2 independent reviewers using a standard data collection form:

1. general information, title, author, and age of the participants;
2. the general circumstances of the subjects, baseline information of each group, and intervention measures administered to each group;
3. clinical outcomes.

Any discrepancies between reviewers were resolved by means of discussion with a third independent researcher. The quality of included studies was assessed according to the improved Jadad scale.

### 2.4 Statistical analysis

We used a Bayesian random effect model to compare all the intervention measures in the NMA directly. A comprehensive evaluation was concurrently performed using the Markov chain Monte Carlo method, which simulates the posterior distribution of the parameters according to the following steps: the model logic and grammar are checked after establishing a model, and data is imported and compiled. During simulation, iteration and annealing were 2000 and 10,000 times, respectively. For the NMA, a Bayesian random effects model was used for analysis, and R software (version 3.3.2) was used to draw graphics to evaluate the model of the convergence. Intervention measures of the network diagram and the node analysis method were applied for consistency identification. In the analysis, 2 groups of events with 0 events were corrected by 0.5.

## 3 Results

### 3.1 Document retrieval

A total of 1621 RCTs were initially retrieved; according to the inclusion and exclusion criteria, a total of 33 RCTs and 8309 patients with AD were selected for further analysis. The MMSE was used as the primary outcome observation index in all the included literature; of the 33 included studies, 30 performed double-arm tests and 3 conducted 3-arm experiments.

### 3.2 The basic features in the study

The basic features included intervention measures, MMSE scores before and after treatment, and the duration of observation (Table 1).

**Table 1:**
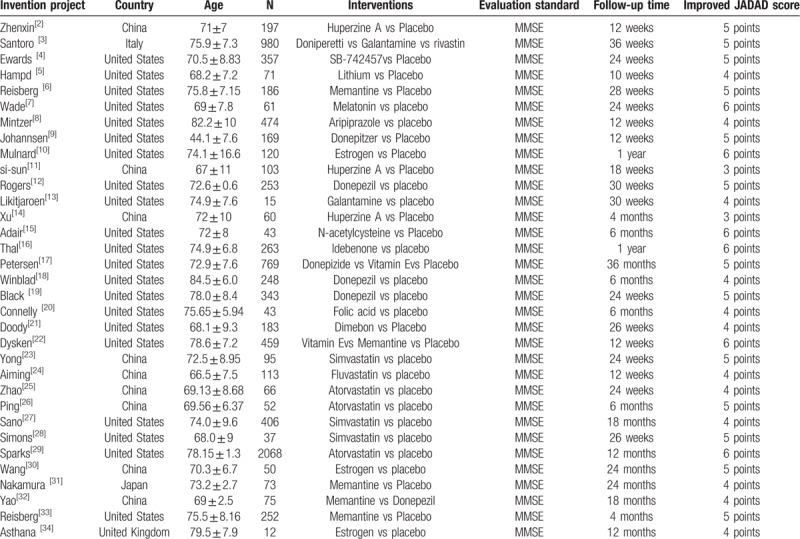
Basic features of the incorporated literature.

### 3.3 Quality evaluation

The baselines of the 33 RCTs were comparable. All reports used MMSE scores to evaluate patient outcomes and, with JADAD scores of 4 to 7, were deemed to be of sufficient quality. The number of individuals and reasons for the loss of visits or shedding were mentioned in all 28 articles. The results of specific evaluations are presented in Table 1.

### 3.4 Evaluation of the effect of anti-dementia drugs for AD patients

#### 3.4.1 NMA

**Figure 1:**
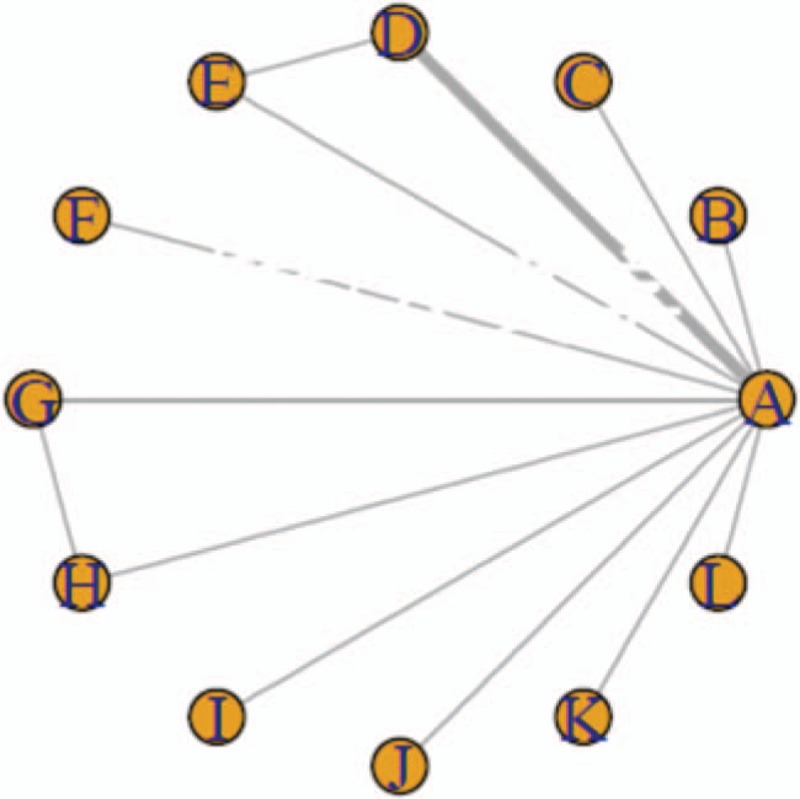
The relationship among interventions to address mild AD-induced cognitive impairment.

**Figure 2:**
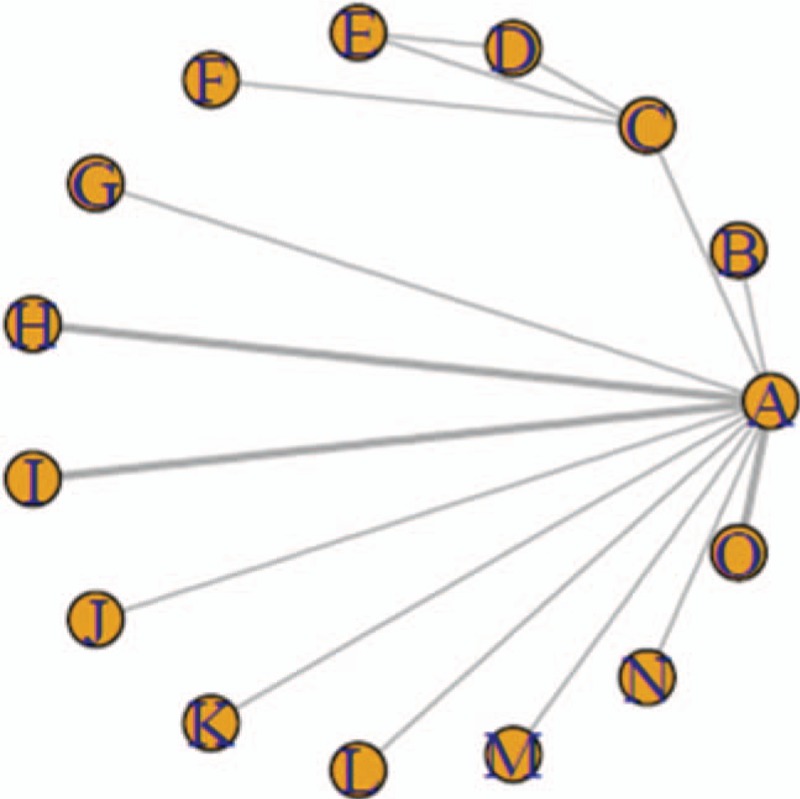
The relationship among interventions to address moderate AD-induced cognitive impairment.

**Figure 3:**
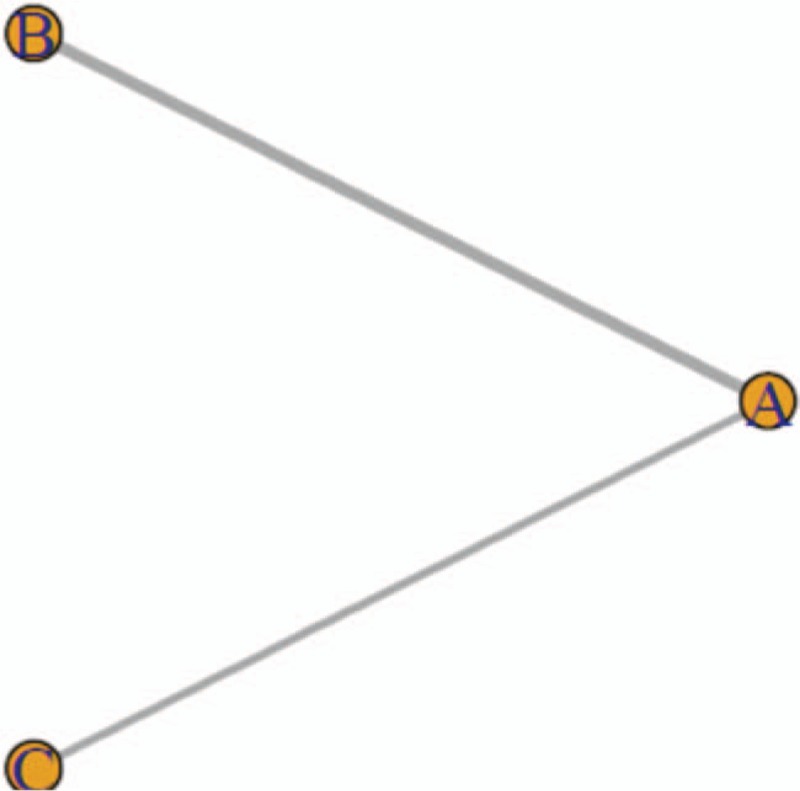
The relationship between interventions to address severe AD-induced cognitive impairment.

The relationship among the interventions administered in the mild, moderate, and severe cognitive impairment subgroups are shown in Figures 1–3, respectively. (Note that lines in the figures denote that there is evidence for a link between 2 interventions. Two interventions that are not directly connected can be indirectly compared by using NMA. The size of a line represents the number of studies.) The results show that, in the subgroup of patients with mild cognitive impairment, the most effective of the 12 drugs administered were donepezil, galantamine, and huperzine A: the mean-difference values were 5.2, 2.5, and 2.4, respectively. There was a direct connection among A, D, E and A, G, H, and an indirect connection among the remaining drugs. In the moderate cognitive impairment subgroups, the most efficacious drugs were found to be donepezil, huperzine A, and rivastigmine: the mean-difference values were 3.8, 2.9, and 3.0, respectively; donepezil was the most effective. A direct connection was found among C, D, and E, while only indirect connections were found among the other drugs. In the subgroup of severe cognitive impairment, donepezil induced greater relief of severe cognitive impairment in patients with AD than did memantine. Our results showed that A, B, and C were indirectly connected.

#### 3.4.2 Convergence rate of NMA for patients with AD treated with anti-dementia drugs

**Figure 4:**
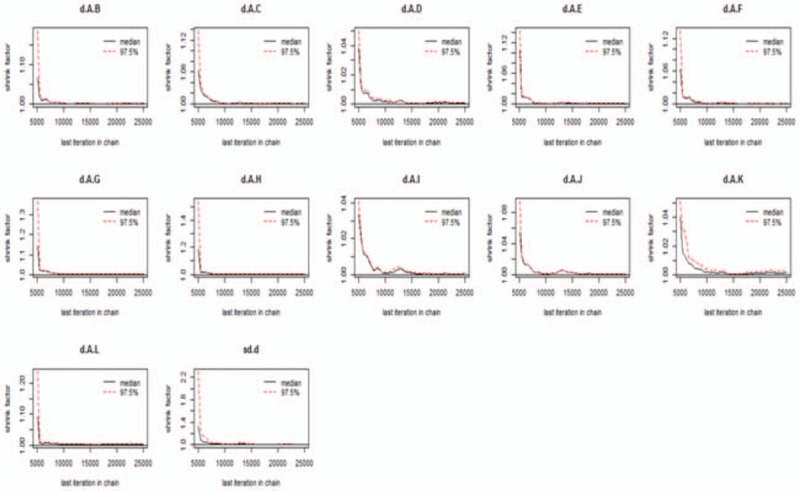
Detection of convergence of mild cognitive impairment.

**Figure 5:**
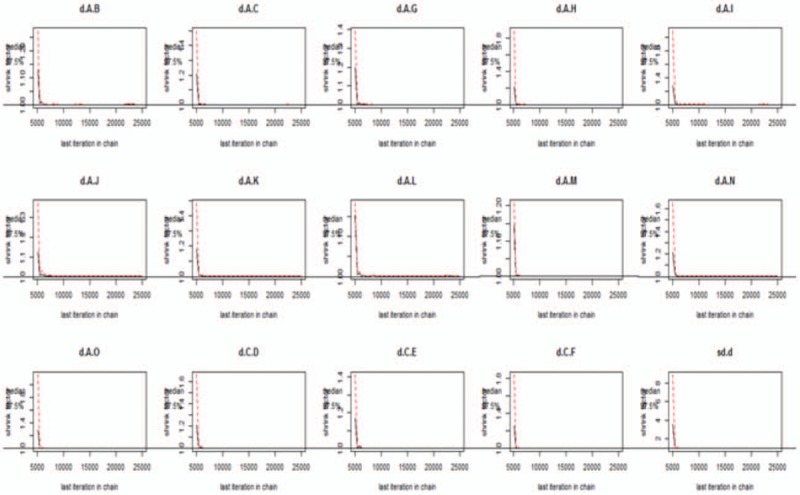
Detection of convergence of moderate cognitive impairment.

**Figure 6:**
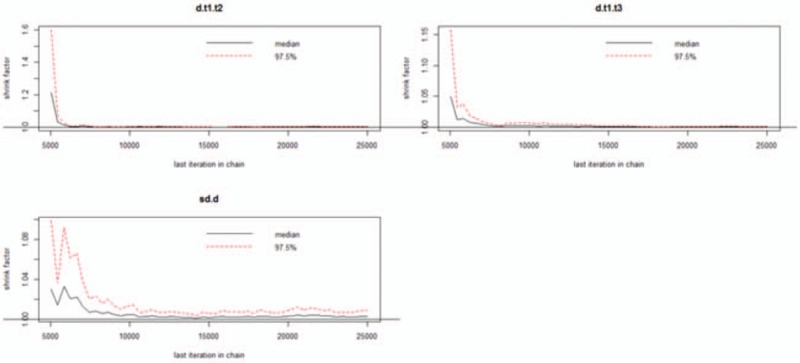
Detection of convergence of severe cognitive impairment.

We used Gemtc software to build models and draw pictures to detect the convergence of the model. The results showed that the subgroups featured good convergence and that the model was reliable (Figs. 4–6).

#### 3.4.3 Conformance analysis of the NMA

**Figure 7:**
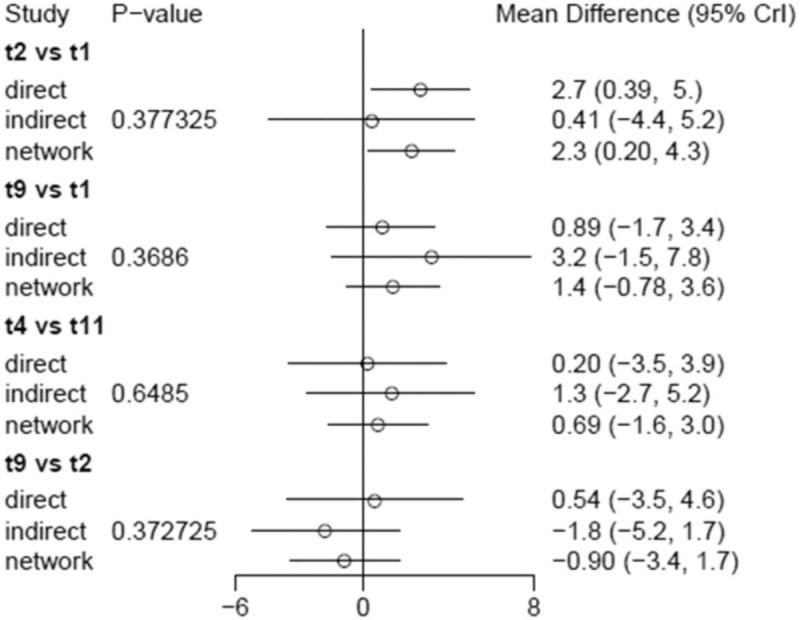
Confirmation of the consistency of the network meta-analysis.

According to the network relation figure, a closed loop could be formed. The conformance test of the network meta-analysis in each subgroup of cognitive dysfunction showed that the line length represents the confidence interval. *P*-values of > .05 indicate good consistency among the reports (Fig. 7).

### 3.5 Donepezil achieved better efficacy in the treatment of patients AD and mild-to-moderate cognitive impairment

**Figure 8:**
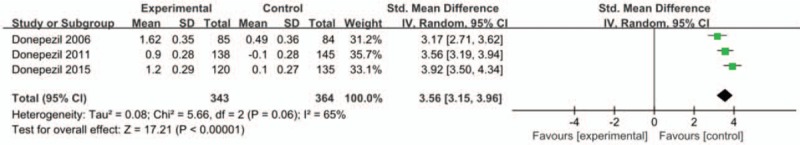
Forest map of the effect of donepezil on patients with AD mild-to-moderate cognitive impairment.

**Figure 9:**
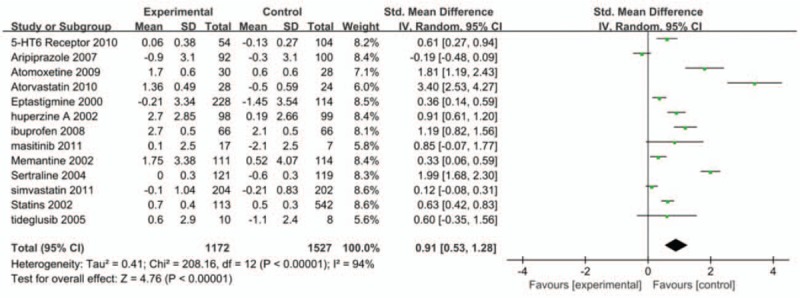
Forest map of the effect of non-donepezil drugs on patients with AD and mild-to-moderate cognitive impairment.

Donepezil was more effective in treating patients with mild or moderate cognitive impairment than the other drugs (Figs. 8 and 9).

### 3.6 No publication bias assessment is found

**Figure 10:**
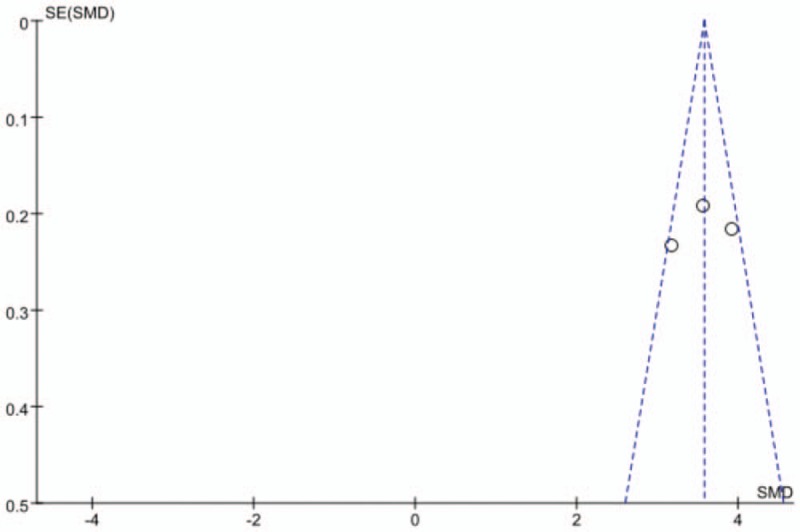
Funnel plot of the effect of donepezil on patients with AD and mild-to-moderate cognitive impairment.

**Figure 11:**
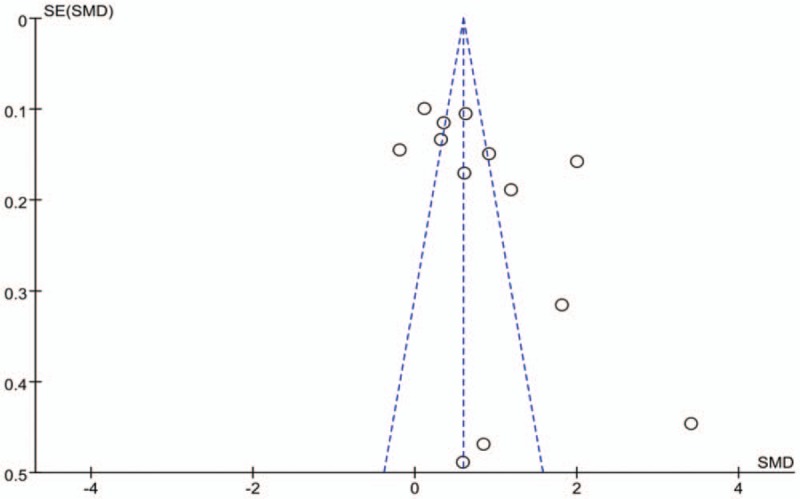
Funnel plot of the effect of non-donepezil drugs on patients with AD and mild-to-moderate cognitive impairment.

Figure 10 shows that all scattered points were found within the funnel plot and formed symmetric distributions at both ends of the dotted line, indicating no publication bias favoring effect of donepezil on alleviating mild-to-moderate cognitive impairment in patients with AD. However, publication bias was found to exists in the use of other drugs to treat patients with AD and mild-to-moderate cognitive impairment (Fig. 11).

## 4 Discussion

Controlling progressive cognitive impairment is an important objective in research on AD treatment. To evaluate the differential effect of anti-dementia drugs according to the degree of cognitive decline exhibited by patients with AD, this NMA divided patients with AD from previous reports into 3 groups according to their respective degrees of cognitive decline as evinced by their MMSE scores: mild, moderate, and severe. Our results indicated that donepezil, galantamine, and huperzine A achieved outstanding efficacy in the mild cognitive function-decline group; this finding agreed with the 2010 European Neurology Union (EFNS)and 2007 American Psychiatric Association (APA)guidelines.^[35,36]^ In the moderate cognitive function-decline group, donepezil, huperzine A, rivastigmine, and galantamine, which are acetylcholinesterase inhibitors, demonstrating the highest efficacy. Kishi ^[37]^ also confirmed that cholinesterase inhibitors could effectively treat mild-to-moderate cognitive impairment in AD patients, and EFNS and APA guidelines recommend acetylcholinesterase inhibitors as a first-line treatment for AD patients with cognitive impairment. Furthermore, we found that donepezil was superior to memantine in slowing severe cognitive impairment in patients with AD. However, Santo et al^[38]^ and Nakamura et al^[39]^ observed memantine was more effective than donepezil in treating non-cognitive symptoms in patients with severe AD and improving their daily behavioral aberrations, such as agitation and delusion. Therefore, while both donepezil and memantine can effectively treat AD with severe cognitive impairment, they address different clinical goals.

In conclusion, this report employed a comprehensive NMA to provide a more intuitive and concise comparison of the efficacies of anti-dementia drugs in the treatment of cognitive impairment. Our review encompassed a total of 18 drugs, including cholinesterase inhibitors, N-methyl-D-aspartate receptor antagonists, anti-oxidation and free radical scavenger drugs, brain metabolism activators, statins, lipid-lowering drugs, and estrogens and among others. We affirmed the superiority of cholinesterase inhibitors, especially donepezil, in the alleviation of cognitive dysfunction in patients with AD irrespective of the degree of impairment. This study therefore may help to inform the clinical choice of anti-dementia drug.

## Author contributions

**Data curation:** Cui-cui CUI, Yong Sun.

**Supervision:** Ying Xing.

**Writing – review & editing:** Xinyi Wang, Yuan Zhang.
